# A new species of *Gaurax* from the Czech Republic (Diptera, Chloropidae)

**DOI:** 10.3897/zookeys.803.29706

**Published:** 2018-12-06

**Authors:** Štěpán Kubík, Miroslav Barták

**Affiliations:** 1 Czech University of Life Sciences, Faculty of Agrobiology, Food and Natural Resources, Department of Zoology and Fisheries, Kamycka 129, 165 00 Praha – Suchdol, Czech Republic Czech University of Life Sciences, Faculty of Agrobiology Praha Czech Republic

**Keywords:** Acalyptratae, diversity, frit flies, new species

## Abstract

*Gauraxsiostrzoneki***sp. n.** (Diptera, Chloropidae) is described from the Czech Republic and the main differential characters are illustrated. A key to the European species of the genus is provided.

## Introduction

The genus *Gaurax* was erected by Loew (1863) for an American species *G.festivus* Loew. Species of the genus *Gaurax* are small black or yellow flies with black markings and with or without ommatrichia, shiny or slightly dusted ocellar triangle, and a rounded scutellum. The gena are usually covered with a silvery dust. The first flagellomere is oval or reniform. The arista are usually densely pubescent, but not thickened. The costal cell of the wing is unusually broad. Legs of males are without a femoral comb. Species of the genus *Gaurax* are variable in body colouration and size, and genitalia examination is usually necessary for species identification.

The larvae of several species are associated with bracket fungi, other fungi, decaying wood infested by insects and decaying vegetable matter, as well as in bird’s nests (Komonen et al. 2004, Nartshuk and Andersson 2013, Nartshuk and Kurina 2014) and several authors before them observed the same. Other species have been reared from cones of *Larixdecidua*, *Piceaabies*, and from a twig of *Pinussilvestris* (Karps 1981; Nordlander and Grijpma 1991).

*Gaurax* is one of the larger genera of the family Chloropidae [for example *Chlorops* includes 343 valid species, *Tricimba* includes 170 valid species, *Gaurax* includes 152 valid species, all from the World database Chloropidae of M. von Tschirnhaus, Bielefeld, cited as von Tschirnhaus in litt.]. It is distributed in all zoogeographical regions. Altogether, 13 described and valid species occur in Europe: *Gauraxborealis* (Duda, 1933), *Gauraxdubius* (Macquart, 1835), *Gauraxephippium* (Zetterstedt, 1848 [= *G.strobilum* Karps, 1981]), *Gauraxfascipes* Becker, 1910, *Gauraxflavomaculatus* (Duda, 1933 [= *G.britannicus* Deeming, 1980]), *Gauraxflavoscutellatus* (Stackelberg, 1955), *Gauraxfungivorus* Nartshuk & Andersson, 2013, *Gauraxgauracicornis* (Duda, 1933), *Gauraxleucarista* Nartshuk, 1962, *Gauraxmaculipennis* (Zetterstedt, 1848), *Gauraxmacrocerus* (Nartshuk, 1962), *Gauraxniger* Czerny, 1906, *Gauraxpolonicus* Nartshuk, 1980, and *Gauraxsuecicus* Nartshuk & Andersson, 2013. Duda (1932–1933) described one more species, *G.gauracicornis*, from Spain. The species was described based on a female only; the male remains unknown and elucidation of the status of this species will be possible only after examination of male genitalia. We did not check if *Oscinellakuntzei* Becker, 1910 is possibly identical with our new species; Nartshuk (1984) lists it as a synonym of *G.maculipennis*, while Duda (1932–1933) treats it as a synonym of *G.dubius*. The male genitalia had never been investigated. Becker (1910) mentioned only one type specimen without sex determination. But Kramer (1917) mentioned a series of both sexes reared from beech mushrooms. A male from this series, not being paratypes, should be studied in the future to confirm the synonymy. One additional species of this genus is described here as new.

## Materials and methods

**Figure 1.**
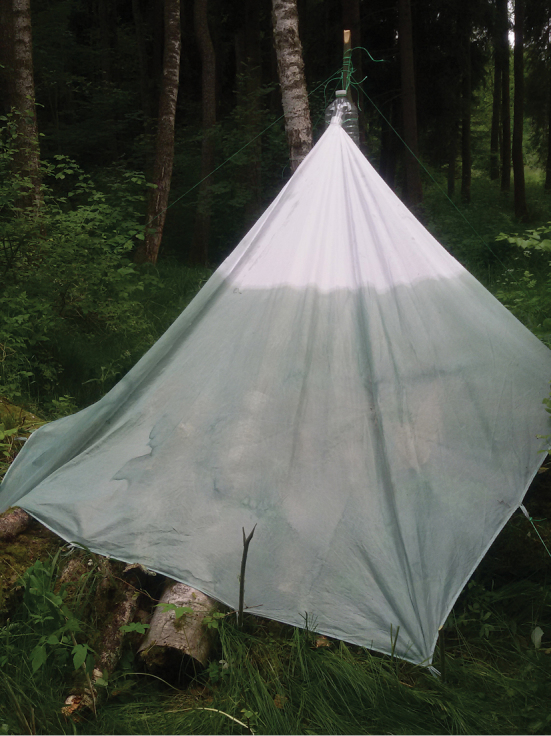
Pyramidal trap.

**Figure 2.**
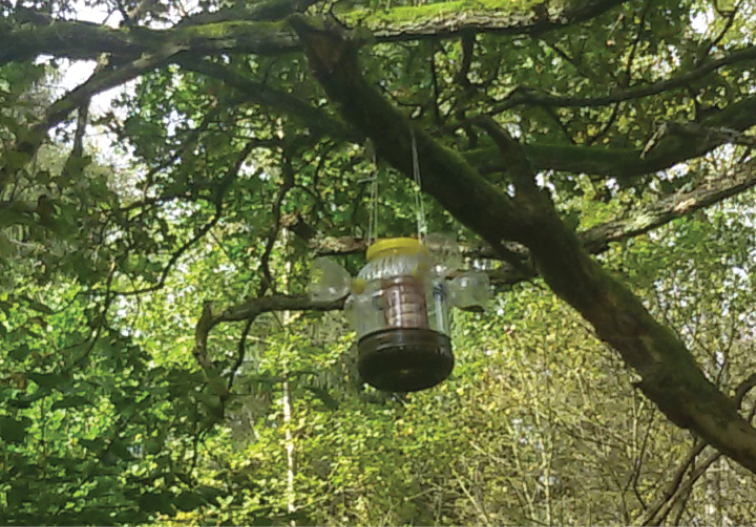
Sawdust trap baited with oak sawdust.

The studied material was collected in 2010 and 2014 by the authors in Vráž (near Písek), and it is deposited in the collections of the Czech University of Life Sciences, Prague. The specimens were collected by pyramidal traps as illustrated in Fig. 1 (described by Barták and Roháček 2011) and by sawdust traps baited with oak sawdust (Fig. 2). Most of the specimens were originally preserved in alcohol and were dried and mounted using the method described by Barták (1997). The genitalia of the described species were macerated in 10 % KOH (24 hours, room temperature) and later stored together with the specimens on plastic tags and fixed with butyl-methacrylate copolymer of methyl-methacrylate, xylene. The genitalia and individual species were photographed using a Nikon D300 digital camera mounted on a Nikon SMZ-U microscope and images were edited with the computer software NIS-Elements 3.0. On average, each final image is from a stack of 15 layers. Images were improved using the software Adobe Photoshop. The genitalia served as models for the outline of the hand drawn illustrations; details were added by direct observation of the genitalia. The morphological terms used here and distribution follow Nartshuk and Andersson (2013). The length of the ocellar triangle was measured from the posterior margin of the posterior ocelli to the apex of the main part of the ocellar triangle. The depth of the head was expressed as the distance between the uppermost part of the head and the lowest part of the gena (in lateral view). The head length was measured from the level of the posterior of the head horizontally to the level of the foremost extension of the anterior margin of frons or eye, excluding the antenna. All measurements (including body length) were taken from dry specimens (therefore the actual length may differ). The body lengths of males were measured from the antennal base to the hind end of the epandrium.

## Results

### Genus Gaurax

Taxon classificationAnimaliaDipteraChloropidae

Loew, 1863

Gaurax Loew, 1863, Berl. ent. Ztschr., 7: 35.

#### Type species.

*Gauraxfestivus* Loew. By monotypy. = *Botanobia* Lioy, 1864, Atti 1st. Veneto Sci. (3) 9: 1125. = *Neogaurax* Malloch, 1914, Canad. Ent., 46 (4): 119.

### Gaurax siostrzoneki sp. n.

Taxon classificationAnimaliaDipteraChloropidae

http://zoobank.org/27B76064-6140-429F-A3B8-BAC34BC13305

**Figure 3.**
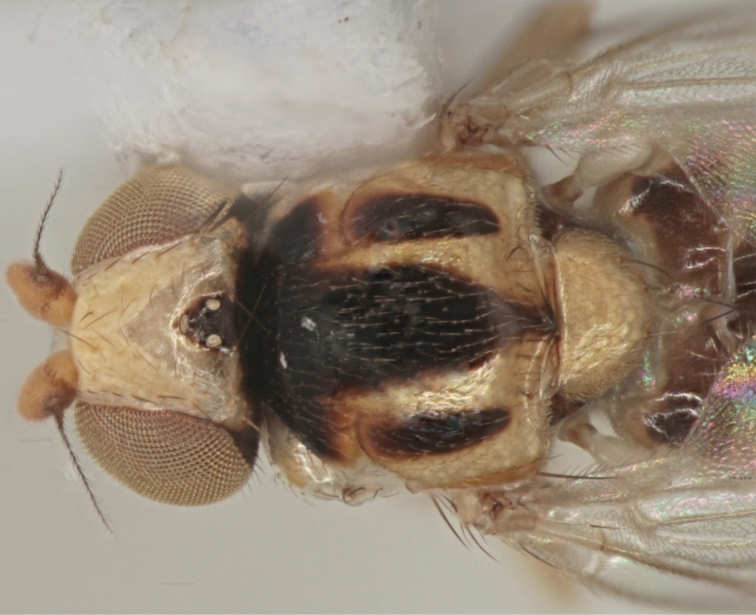
*Gauraxsiostrzoneki* sp. n. (paratype): body, dorsal view.

**Figure 4.**
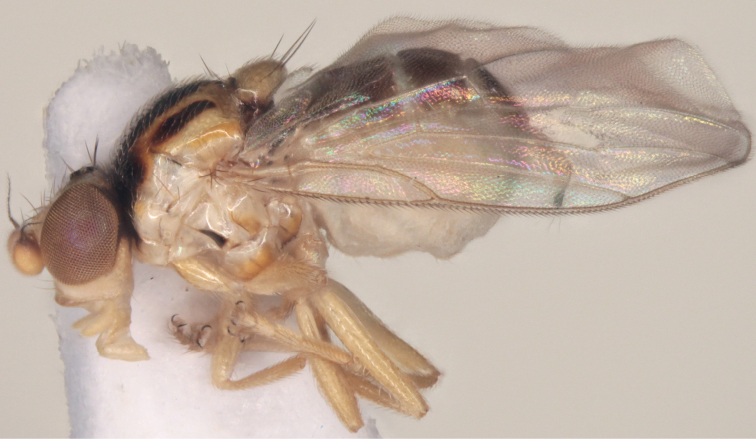
*Gauraxsiostrzoneki* sp. n. (paratype): body, lateral view.

**Figure 5.**
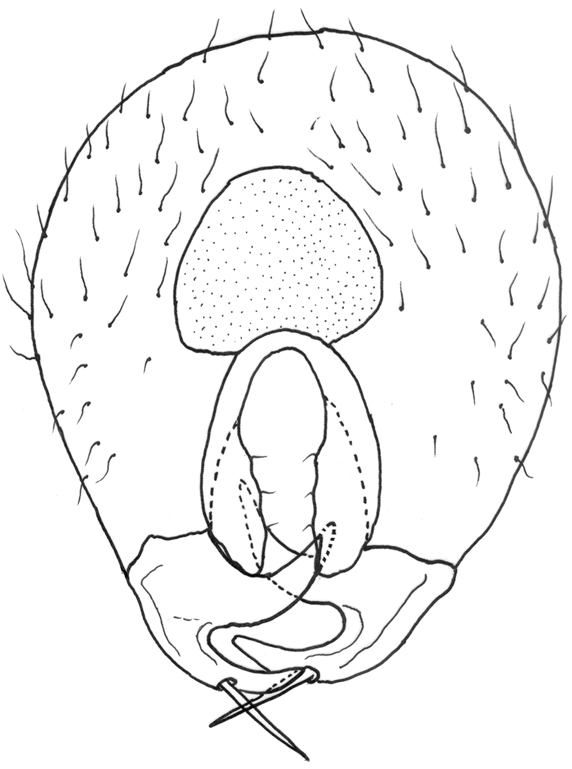
*Gauraxsiostrzoneki* sp. n. (holotype): epandrium posterior view.

**Figure 6.**
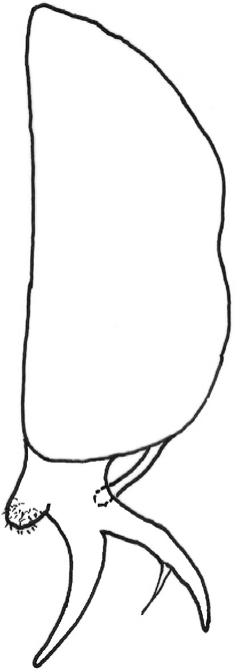
*Gauraxsiostrzoneki* sp. n. (holotype): epandrium lateral view.

Figures 3 , 4 , 5 , 6

#### Holotype male.

**Czech Republic, Bohemia**, Vráž nr. Písek, alder forest, 430 m, PyrT [= pyramidal trap], 49°24'8"N/14°7'8"E, 25.vi–19.vii.2010, Barták leg. Holotype is in good condition, abdomen on plastic tag together with the specimen. **Paratypes**. 3 males, same data as the holotype, 4 males and 4 females: Vráž nr. Písek, 400 m, Sawdust trap, 49°24'12"N/14°7'3"E, 12.vi–30.ix.2014, Barták leg.

#### Diagnosis.

Species with head 1.25× as deep as long, first flagellomere 1.3× as deep as long, body yellow with three black partly fused stripes, central stripe reaching scutellum and scutellum often dark. *Gauraxsiostrzoneki* sp. n. is similar to *G.flavoscutellatus*. The main characters distinguishing these two species are as follows: *Gauraxsiostrzoneki* has surstylus with two long curved extensions, one long, strong seta growing from the lower projection, and cercus short and enlarged at apical part. In *G.flavoscutellatus* surstylus is without spurs and cercus is short and narrowed.

#### Description.

*Male* (Figs 3, 4). *Body* length 1.3–1.5 mm. Ground colour yellow. Head 1.25× as deep as long (lateral view), yellow with black occiput. Ocellar triangle occupying two-thirds of frons, yellow, shiny, and black on ocellar tubercle only, with one row of dark interfrontal setulae along sides. All setae and setulae on head black. First flagellomere 1.3× as deep as long, yellow, often darkened on outer margin (variable). Arista black. Eyes with ommatrichia. Depth of gena in front equal to the length of first flagellomere, gena with one row of dark setulae. Palpus yellow.

*Thorax.* Scutum shiny yellow with three black partly fused stripes, central stripe reaching scutellum, sometimes scutum completely dark. Scutellum yellow or dark, apical scutellar setae longer than lateral setae. Anepisternum, katepisternum, katepimeron, and meron shiny yellow with 1-4 small dark spots. Notopleural setae 1+1. Setae of thorax black. Wings not coloured. Haltere yellow. Legs completely yellow.

*Abdomen*: black dorsally and yellow ventrally. Male genitalia (Figs 5, 6): epandrium yellow with dark, wide, medial stripe, surstylus with two long curved extensions, one long, strong seta growing from the lower projection, cercus short and enlarged at apical part.

#### Etymology.

Named in honour of Archbishop of Břevnov Monastery Petr Prokop Siostrzonek, a supporter of natural science and our friend.

### The key of European species of *Gaurax* Loew (modified from Nartshuk and Andersson 2013)

**Table:** 

1	First flagellomere black with white pubescence; arista white, slightly thickened	***G.leucarista* Nartshuk**
–	First flagellomere and arista different	**2**
2	Head depth equal to length (Fig. 7)	**3**
–	Head 1.25× as deep as long (Fig. 8)	**12**
3	First flagellomere rounded, nearly as deep as long	**4**
–	First flagellomere reniform, 1.3× as deep as long	**6**
4	First flagellomere large, nearly as deep as height of face	***G.macrocerus* (Nartshuk)**
–	First flagellomere smaller, narrower than height of face	**5**
5	Male genitalia: cercus narrow, rather long; surstylus strongly elongated, widened apically (Fig. 9). Body mainly, sometimes entirely black	**G. borealis (Duda)**
–	Male genialia: cercus shorter and wider, surstylus shorter (Fig. 10). Colour of body variable, but thorax usually yellow with black stripes fused on anterior part of scutum, scutellum yellow, pleuron with four black spots	***G.dubius* (Macquart)**
6	Apical part of wing slightly darkened especially in male	***G.maculipennis* (Zetterstedt)**
–	Wing without any darkening	**7**
7	Ocellar triangle yellow, shiny, only on ocellar tubercle black	**8**
–	Ocellar triangle mainly or entirely black, shiny or dusted	**9**
8	All legs yellow. Dark band on hind tibia equals one quarter of tibia length	***G.fascipes* Becker**
–	All legs yellow. Dark band on hind tibia equals one halfr of tibia length	***G.polonicus* Nartshuk**
9	Body mainly black except yellow front margin of frons and gena, or also scutellum, notopleuron, and hind part of postpronotum yellow, legs darkened or yellow with black mark	**10**
–	Body usually yellow with dark stripes on scutum. Frons, genae, and scutellum yellow. Legs yellow	**11**
10	Body mainly black except yellow front margin of frons and gena. All legs darkened	***G.suecicus* Nartshuk & Andersson**
–	Body black, notopleuron, hind part of postpronotum and upper part of anepisternum yellow. Legs yellow with black mark on all femora and mid and hind tibiae	***G.flavomaculatus* (Duda)**
11	Male genitalia: cerci tapering, close to each other, surstylus with acute process directed medially	***G.fungivorus* Nartshuk & Andersson**
–	Male genitalia: cerci broader and wider apart, surstylus with several processes on lower margin (Fig. 11)	***G.ephippium* (Zetterstedt)**
12	Body completely black	***G.niger* Czerny**
–	Body yellow with three black stripes on scutum, often partly or completely fused	**13**
13	Ocellar triangle mostly black with yellow edge and partly dusted, male genitalia as in Fig. 12	***Gauraxflavoscutellatus* (Stackelberg)**
–	Ocellar triangle yellow, shiny and black on ocellar tubercle only, male genitalia as in Figs 5, 6	***G.siostrzoneki* sp. n.**

**Figure 7.**
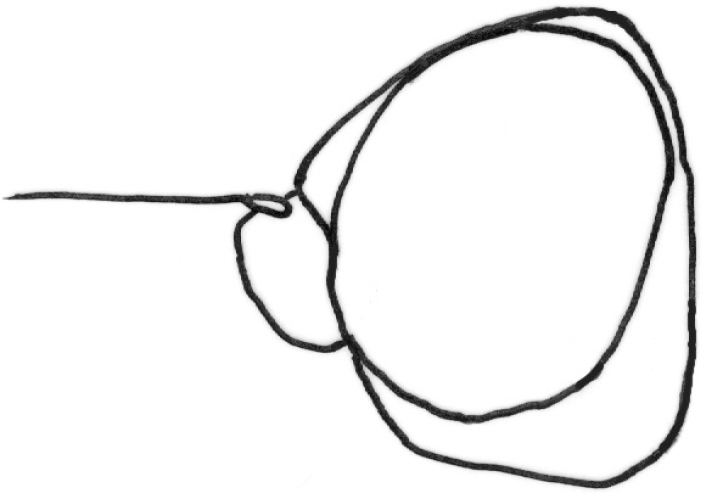
Couplet 2a of key, head, lateral view (after Nartshuk and Andersson 2012).

**Figure 8.**
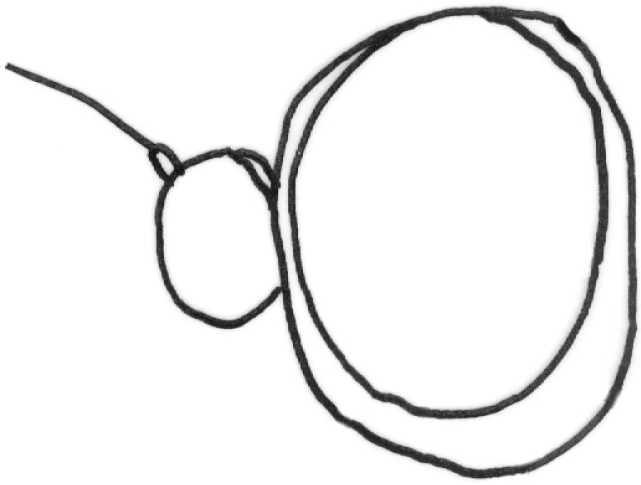
Couplet 2b of key, head, lateral view (after Nartshuk et al. 1970).

**Figure 9.**
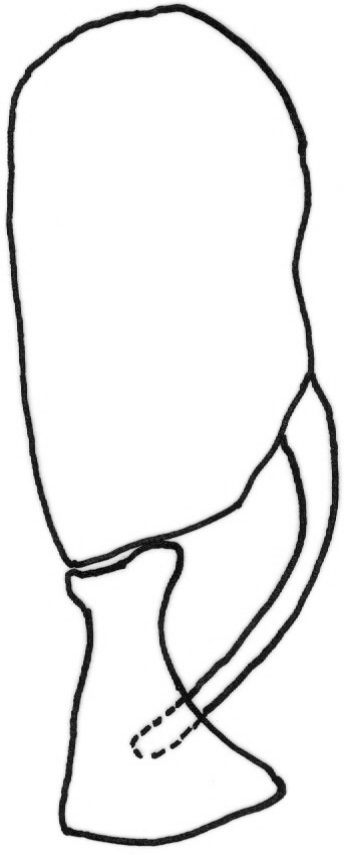
*Gauraxborealis*: epandrium lateral view (after Nartshuk and Andersson 2013).

**Figure 10.**
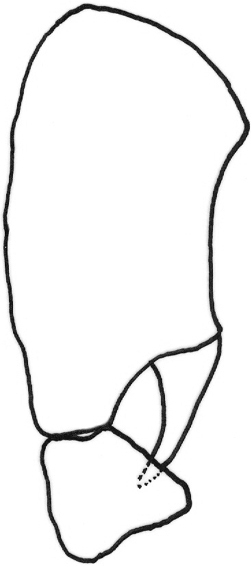
*Gauraxdubius*: epandrium lateral view (after Nartshuk and Andersson 2013).

**Figure 11.**
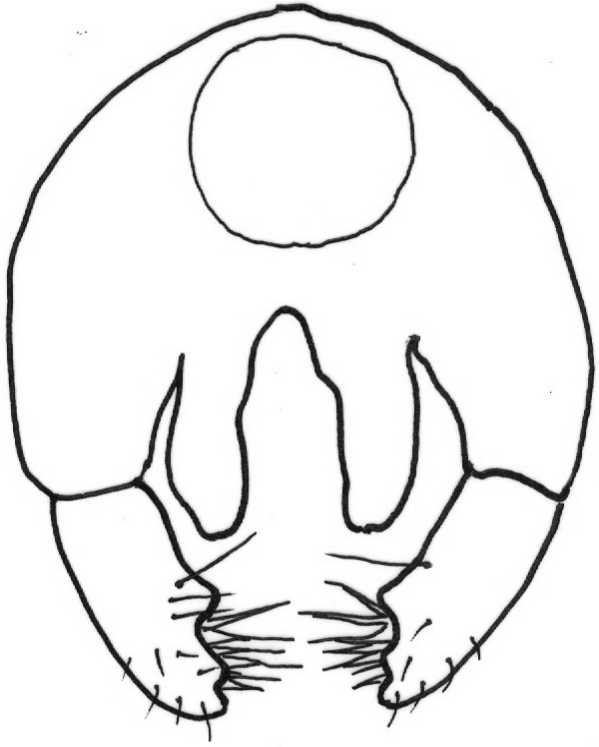
*Gauraxephippium*: epandrium posterior view (after Nartshuk and Andersson 2013).

**Figure 12.**
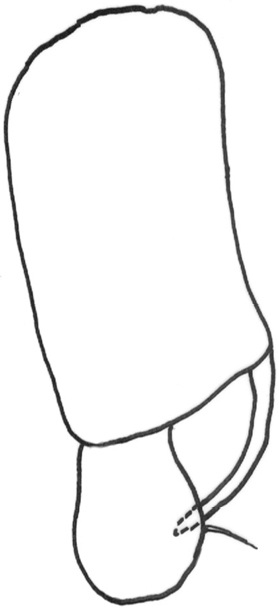
*Gauraxflavoscutellatus*: epandrium lateral view.

## Supplementary Material

XML Treatment for Gaurax

XML Treatment for Gaurax siostrzoneki
